# Hydrocortisone treatment is associated with a longer duration of MODS in pediatric patients with severe sepsis and immunoparalysis

**DOI:** 10.1186/s13054-020-03266-x

**Published:** 2020-09-04

**Authors:** Katherine E. Bline, Melissa Moore-Clingenpeel, Josey Hensley, Lisa Steele, Kristin Greathouse, Larissa Anglim, Lisa Hanson-Huber, Jyotsna Nateri, Jennifer A. Muszynski, Octavio Ramilo, Mark W. Hall

**Affiliations:** 1 The Abigail Wexner Research Institute at Nationwide Children’s Hospital, Columbus, OH USA; 2 Division of Critical Care Medicine, Nationwide Children’s Hospital, 700 Children’s Drive, Columbus, OH 43205 USA; 3 Biostatistics Resource at Nationwide Children’s Hospital, Columbus, OH USA; 4 Division of Infectious Diseases, Nationwide Children’s Hospital, Columbus, OH USA

**Keywords:** Sepsis, Pediatric, Hydrocortisone, Shock, Immunoparalysis, MODS, Immune

## Abstract

**Background:**

Severe critical illness-induced immune suppression, termed immunoparalysis, is associated with longer duration of organ dysfunction in septic children. mRNA studies have suggested differential benefit of hydrocortisone in septic children based on their immune phenotype, but this has not been shown using a functional readout of the immune response. This study represents a secondary analysis of a prospectively conducted immunophenotyping study of pediatric severe sepsis to test the hypothesis that hydrocortisone will be differentially associated with clinical outcomes in children with or without immunoparalysis.

**Methods:**

Children with severe sepsis/septic shock underwent blood sampling within 48 h of sepsis onset. Immune function was measured by quantifying whole blood ex vivo LPS-induced TNFα production capacity, with a TNFα response < 200 pg/ml being diagnostic of immunoparalysis. The primary outcome measure was number of days in 14 with MODS. Univariate and multivariable negative binomial regression models were used to examine associations between hydrocortisone use, immune function, and duration of MODS.

**Results:**

One hundred two children were enrolled (age 75 [6–160] months, 60% male). Thirty-one subjects received hydrocortisone and were more likely to be older (106 [52–184] vs 38 [3–153] months, *p* = 0.04), to have baseline immunocompromise (32 vs 8%, *p* = 0.006), to have higher PRISM III (13 [8–18] vs 7 [5–13], *p* = 0.0003) and vasoactive inotrope scores (20 [10–35] vs 10 [3–15], *p* = 0.0002) scores, and to have more MODS days (3 [1–9] vs 1 [0–3], *p* = 0.002). Thirty-three subjects had immunoparalysis (TNFα response 78 [52–141] vs 641 [418–1047] pg/ml, *p* < 0.0001). Hydrocortisone use was associated with longer duration of MODS in children with immunoparalysis after adjusting for covariables (aRR 3.7 [1.8–7.9], *p* = 0.0006) whereas no association with MODS duration was seen in children without immunoparalysis (aRR 1.2 [0.6–2.3], *p* = 0.67).

**Conclusion:**

Hydrocortisone use was independently associated with longer duration of MODS in septic children with immunoparalysis but not in those with more robust immune function. Prospective clinical trials using a priori immunophenotyping are needed to understand optimal hydrocortisone strategies in this population.

## Background

Septic shock is a leading cause of morbidity and mortality in children, with over 7000 pediatric deaths due to sepsis in the USA each year [1, 2]. Septic shock is characterized by a dysregulated systemic immune response to infection that results in organ dysfunction. The failure of more than one organ, termed “multiple organ dysfunction syndrome (MODS),” confers a > 10-fold increase in risk of mortality in critically ill children [3]. Current management guidelines for pediatric sepsis largely focus on supportive care, including fluid resuscitation and vasoactive medications, in addition to early empiric antibiotics [4]. Hydrocortisone is frequently prescribed as an adjunctive treatment for children with septic shock who have known adrenal insufficiency, those with a history of recent corticosteroid use, and those who remain hemodynamically unstable despite fluid resuscitation and initiation of vasoactive support [5, 6]. The use of hydrocortisone in septic shock, however, remains controversial, with clinical trials in adults yielding conflicting results [7–9]. The current version of the pediatric Surviving Sepsis guidelines is unable to recommend for or against hydrocortisone use, largely due to a lack of evidence. The first prospective clinical trial of hydrocortisone use in pediatric septic shock is currently underway (NCT03401398).

The immune response to pediatric critical illness is highly dynamic, with acquired immune suppression frequently accompanying systemic inflammation. When severe, this compensatory immune suppression is termed “immunoparalysis.” We and others have consistently shown that immunoparalysis is associated with adverse outcomes from critical illness in children including sepsis, trauma, and cardiopulmonary bypass [10–13]. We recently observed, in a prospective, single-center, 102-subject cohort of children with severe sepsis/septic shock, that both immunoparalysis and hydrocortisone treatment were associated with longer duration of organ dysfunction. Prior transcriptomic work has suggested the possibility of a differential effect of hydrocortisone on pediatric sepsis outcomes depending on the host immune phenotype [14]. We therefore designed this secondary analysis of our data set to test the hypothesis that the relationships between hydrocortisone treatment and duration of MODS will be variable, depending on the presence or absence of immunoparalysis.

## Methods

### Study population

This is a secondary analysis of a prospective observational study which was conducted in the 54-bed medical-surgical pediatric intensive care unit (PICU) at Nationwide Children’s Hospital, a quaternary-care children’s hospital with over 3000 annual admissions. Children < 18 years of age who were admitted to the PICU were eligible for enrollment if they met consensus criteria for severe sepsis or septic shock [15] within the preceding 48 h. Subjects were excluded if a limitation-of-care order was in place, if there was expected progression to brain death by the primary treating team, or if they were admitted to the cardiothoracic ICU. Written informed consent was obtained from subjects’ legal guardians prior to participation, and if appropriate, subjects’ assent was obtained. The protocol was approved by the Institutional Review Board at Nationwide Children’s Hospital.

### Immune function

Whole blood samples were collected within 48 h of sepsis onset. Complete blood count (CBC) testing was done as part of routine care by the primary treating team, and all subjects had a CBC with differential completed within the first 48 h of sepsis onset. If multiple CBCs were obtained, the absolute cell counts reported are the lowest values within the first 48 h. Plasma from un-stimulated blood samples was collected after centrifugation of whole blood at 1000×*g* for 5 min and stored at − 80 °C to quantify interleukin (IL)-10 and IL-6, reflecting the systemic inflammatory response. Plasma TNFα levels were not measured, as previous studies have demonstrated that circulating TNFα comprises less than 10% of the measured ex vivo TNFα production capacity [16].

Innate immune function was measured by whole blood ex vivo lipopolysaccharide (LPS)-induced tumor necrosis factor (TNF)-α production capacity. Briefly, 50-μl aliquots of heparinized whole blood were added to stimulation tubes containing LPS (500 pg/ml, phenol-extracted from *Salmonella abortus equi* [Sigma, St. Louis, MO]). Stimulation tubes were then incubated at 37 °C for 4 h. After incubation, supernatants were collected and stored at − 80 °C for batched cytokine analysis. TNFα from LPS-stimulated supernatants were quantified by the *Immulite 1000* automated chemiluminometer (Siemens Healthcare Diagnostics, Deerfield, IL). Immunoparalysis was defined as an LPS-induced TNFα production capacity (hereafter referred to as the TNFα response) < 200 pg/ml [17, 18].

### Clinical data and outcome measurements

For subjects with community-acquired sepsis, sepsis onset was defined as the initial time of presentation to any emergency department that resulted in hospitalization. For subjects who developed nosocomial sepsis, sepsis onset was defined as the time of transfer to the PICU. The electronic medical record was used to collect clinical data. Baseline immune compromise was defined as the presence of a congenital immunodeficiency, an oncologic diagnosis, or the receipt of chronic immunosuppressive therapy. Complex chronic conditions were defined as previously published [19]. The highest Pediatric Risk of Mortality (PRISM) III score within the first 24 h of sepsis onset was used to measure initial illness severity. The highest vasoactive inotrope score (VIS) in the first 48 h, which is calculated using the combined doses of inotropic and vasopressor infusions [20], was used as a surrogate measure of subjects’ shock state.

MODS was defined as dysfunction of more than one organ system according to previously published criteria [21] and represented only new organ dysfunction, not chronic organ dysfunction. The primary outcome measure was the number of days with MODS in the first 14 days from sepsis onset. Non-survivors were assigned the maximum number of MODS days (fourteen).

### Statistical analysis

Values are reported as median and interquartile range (IQR) or *n* and % as appropriate. Simple comparisons were made using chi-square or Fisher’s exact tests for categorical variables and Wilcoxon rank-sum tests for continuous variables. Univariate and multivariable negative binomial regression models were used to examine associations with duration of MODS. Coefficients for all negative binomial regression models were exponentiated to reflect risk ratios, or the percent increase in duration of MODS for a unit increase in the given predictor variable. Variable selection for the multivariable models was based on stepwise selection with an entry criterion of *p* < 0.15 and an exit criterion of *p* > 0.1, in addition to Akaike’s Information Criterion (AIC, a measure of goodness of fit); VIS, baseline immune compromise, and hydrocortisone were retained in all models regardless of statistical significance on clinical grounds. To explore the possibility that an association between hydrocortisone use and outcomes may simply reflect the initial shock state of the subject, we performed secondary analysis limited to the subgroup of subjects with septic shock (those treated with vasoactive drugs). Statistical analyses were performed using SAS 9.4 (SAS Institute, Cary, NC) and Prism 7.0 (GraphPad Software, La Jolla, CA, USA).

## Results

### Subjects

A total of 102 subjects were enrolled between January 2012 and April 2014 with a median age of 75 (6–160) months. Subject demographic and clinical characteristics are shown in Table 1. Forty-eight patients had a complex chronic condition, most commonly including diagnoses of developmental delay or seizure disorder. There were 16 patients with baseline immune compromise due to receipt of solid organ or bone marrow transplant (*n* = 5), oncologic diagnosis (*n* = 6), congenital immunodeficiency (*n* = 2), or receipt of immunosuppressive medications for autoimmune disease (*n* = 3). Thirty-one subjects (30%) received hydrocortisone. Of those, 14 (45%) were known to have adrenal insufficiency, recent steroid use, or evidence of adrenal insufficiency with a cortisol level < 18 μg/dl in the setting of sepsis. Hydrocortisone-treated subjects were more likely to be older and to have baseline immune compromise, as well as a higher PRISM compared to those that did not receive hydrocortisone (Table 1). In the entire cohort, subjects that received hydrocortisone had a longer duration of MODS compared to those who did not receive hydrocortisone (3 [1–9] vs 1 [1–3] days, *p* = 0.0002). In the subset with septic shock, this relationship was similar (3.5 [2–9] vs 2 [0–3] days, *p* < 0.0001). All subjects who died were treated with hydrocortisone. There was no difference in the incidence of nosocomial infection between subjects treated with or without hydrocortisone in the cohort as a whole (2/31 [6%] vs 8/71 [11%], *p* = 0.72) or in the subgroup with septic shock (1/26 [4%] vs 5/54 [9%], *p* = 0.66).

**Table 1:** Cohort characteristics

Characteristics	All (***N*** = 102)		No hydrocortisone (***N*** = 71)		Hydrocortisone (***N*** = 31)		***p*** value
	Median or ***N***	IQR or %	Median or ***N***	IQR or %	Median or ***N***	IQR or %	
Age, months	74.5	(6, 160)	38	(3, 153)	106	(52, 184)	**0.0441**
Male	61	60	40	56	21	68	0.2799
Complex chronic condition	48	47	29	41	19	62	0.0571
Baseline immune compromise	16	16	6	8	10	32	**0.0057**
RBC transfusion in first 48 h	48	47	21	44	17	55	0.2983
Acute comorbidities	18	18	14	20	4	13	0.4063
PRISM	9	(5, 14)	7	(5, 13)	13	(8, 18)	**0.0003**
PELOD*	7	(5, 9)	6	(5, 9)	8	(5, 11)	0.0813
VIS	11.5	(5, 20)	10	(3, 15)	20	(10, 35)	**0.0002**
Mortality	6	6	0	0	6	19	**0.0005**
*Immune function:*							
Plasma IL6* (pg/mL)	78.85	(22.7, 258)	63.9	(24, 140)	112	(17.9, 811)	0.2916
Plasma IL10* (pg/mL)	30.55	(10, 85.45)	21.5	(10, 75.9)	35.9	(11.4, 95.7)	0.3586
Ex vivo TNFα^#^ (pg/mL)	433	(143.5, 821)	465	(237.5, 956.5)	180.5	(52.3, 633)	**0.0063**
AMC^#^ (cells/mm3)	326.5	(93, 648)	320	(93, 728)	412	(75, 545)	0.6675

*RBC* red blood cell, *PRISM* Pediatric Risk of Mortality Score, *PELOD* Pediatric Logistic Organ Dysfunction, *AMC* absolute monocyte count

*Highest value in first 48 h

^#^Lowest value in first 48 h

In multivariable analysis, the receipt of hydrocortisone in the first 48 h, the presence of a complex chronic condition, and receipt of a red blood cell transfusion in the first 48 h were all independently associated with a higher adjusted relative risk (aRR) for longer duration of MODS in the complete cohort (Table 2). In the subset of subjects with septic shock, there was an even stronger independent association between hydrocortisone use and longer duration of MODS.

**Table 2:** Adjusted risk of longer duration of MODS in entire cohort and those with septic shock

Variable	Entire cohort				Septic shock subset			
	aRR	Lower CL	Upper CL	p-value	aRR	Lower CL	Upper CL	***p*** value
Baseline immune compromise	0.95	0.49	1.85	0.891	0.56	0.28	1.14	0.1117
CCC	1.97	1.22	3.19	**0.0054**	1.73	1.09	2.76	**0.0198**
VIS	1.01	1.00	1.03	**0.0166**	1.01	1.00	1.02	0.1236
RBC Transfusion	1.83	1.18	2.83	**0.0066**	1.47	0.94	2.28	0.0882
Hydrocortisone	1.79	1.06	3.04	**0.0295**	2.74	1.60	4.70	**0.0002**

*CCC* complex chronic condition, *VIS* Vasoactive Inotrope Score, *RBC* red blood cell

### Hydrocortisone and immune function

**Fig. 1:**
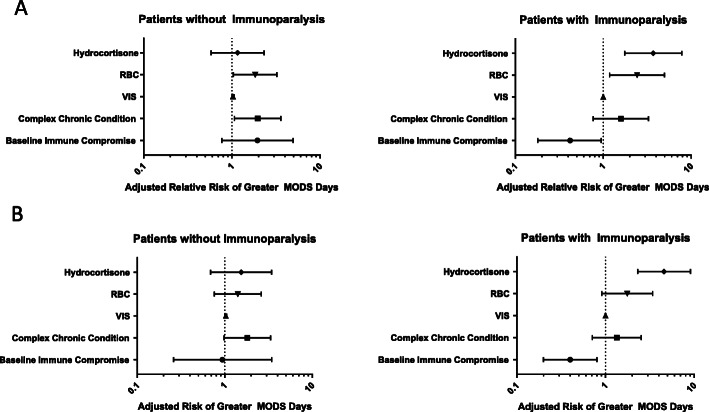
Forest plot of the adjusted relative risk of variables on duration of MODS in the total cohort (**a**) and the subjects with septic shock (**b**). In both the total cohort and septic shock subset, subjects with immunoparalysis had a higher risk of a greater duration of MODS when receiving hydrocortisone treatment (aRR 3.72 [1.76, 7.87] and aRR 4.57 [2.31, 9.07], respectively)

All subjects underwent immune function testing during the first 48 h of sepsis, though 22/31 (70%) of the subjects in the hydrocortisone-treated group had blood sampling done after receiving at least one dose of hydrocortisone (median of 15.4 h). In the cohort as a whole, subjects in the hydrocortisone-treated group had lower TNFα responses and lower absolute lymphocyte counts compared to subjects not treated with hydrocortisone (*p* = 0.0063 and 0.0027 respectively) (Table 1). The degree of systemic inflammation, as evidenced by plasma IL-6 and IL-10 levels, was similar between groups. Among subjects that received hydrocortisone, immune function and plasma cytokine levels were similar between subjects whose blood sample was obtained before versus after their first dose of hydrocortisone (TNFα response: 247 (65–408) vs 172 (52–545) pg/mL, *p* = 0.86; IL-6: 167 (16–376) vs 70 (18–3214) pg/mL, *p* = 0.533; IL-10: 36 (15–76) vs 45 (13–116) pg/mL, *p* = 0.675). After adjusting for covariables, hydrocortisone treatment in the first 48 h of sepsis was independently associated with a longer duration of MODS in subjects with immunoparalysis (aRR = 3.72, 95% CI 1.76–7.87, *p* = 0.0006) (Fig. 1a). This was not true for subjects without immunoparalysis (aRR = 1.16, 95% CI 0.58–2.32, p = 0.67). This relationship was even stronger in the septic shock subgroup (Fig. 1b), with hydrocortisone use being associated with a longer duration of MODS only in subjects with immunoparalysis (aRR = 4.57, 96% CI 2.31–9.07, *p* < 0.0001 vs aRR = 1.54, 95% CI 0.69–3.43, *p* = 0.29).

## Discussion

Ours is the first study to examine the relationships between the functional immune response, hydrocortisone use, and clinical outcomes in septic children. We were able to identify differential risk for prolonged organ dysfunction in children treated with hydrocortisone depending on their immune function in the acute phase of sepsis, with children with immunoparalysis demonstrating more prolonged MODS. Our data are in agreement with previously published mRNA studies [14] and highlight the need to incorporate prospective immunophenotyping into the design of future clinical trials of hydrocortisone in septic children.

Severe sepsis/septic shock remains a major source of morbidity and mortality worldwide. A recent multi-national point prevalence study found a 25% mortality associated with pediatric severe sepsis [22] while another US multi-center study found a strong correlation between higher organ dysfunction scores and lower health-related quality of life in pediatric survivors of septic shock [23]. The mainstays of pediatric sepsis management include early fluid resuscitation, timely antibiotic administration, and hemodynamic support [6, 24, 25] though hydrocortisone is frequently used as adjuvant therapy in children with fluid and catecholamine-resistant shock as well as those with a priori risk factors for adrenal insufficiency. Hydrocortisone use in sepsis remains highly controversial, however. Evidence suggests that low-dose hydrocortisone treatment is associated with a shorter time to shock reversal in septic adults [26], but its reported effects on mortality have been inconsistent and at least one study has suggested an increase in nosocomial infection risk with hydrocortisone use [8]. Retrospective, observational pediatric data raise the possibility of equivocal or even harmful effects of hydrocortisone in the treatment of sepsis [27–29], but data from pediatric randomized controlled trials are, as yet, lacking. The current version of the pediatric Surviving Sepsis guidelines recommends neither for nor against the use of hydrocortisone [4].

While it is used in septic patients primarily for its hemodynamic-supporting mineralocorticoid activity, hydrocortisone does have some glucocorticoid activity which has the potential to exacerbate or perpetuate sepsis-induced immune suppression. The host immune response to sepsis is highly dynamic. The initial pro-inflammatory response is quickly accompanied by a compensatory downregulation of systemic immune function. This is termed “immunoparalysis” when severe and is characterized by a marked reduction of the ability of whole blood to produce TNFα upon ex vivo stimulation with LPS. We and others have repeatedly shown associations between reduction in the TNFα response and adverse outcomes including nosocomial infection, prolonged organ dysfunction, and death in critically ill children [13, 16, 18]. We recently published the results of a single-center, prospective immune phenotyping study of 102 children with severe sepsis/septic shock in which the use of hydrocortisone and severe reduction in the TNFα response were both associated with longer durations of MODS [17]. The degree to which hydrocortisone influences the host immune response in this setting is unknown. The current study represents a secondary analysis of this cohort, with emphasis on the relationships between hydrocortisone use, immunoparalysis, and MODS.

Our observation that hydrocortisone use was associated with a differential outcome depending on the subject’s immunophenotype is complementary to a recent biomarker study conducted by Wong et al. Their group developed a panel of 5 plasma proteins (the PERSEVERE panel) that has been validated to risk-stratify children with acute septic shock [30]. They also developed a 100-gene leukocyte transcriptomic panel that segregates children with acute septic shock into endotypes that are characterized by under-activation (endotype A) or overactivation (endotype B) of genes related to adaptive immunity and glucocorticoid receptor signaling [31]. In a secondary analysis of 288 children with acute septic shock, subjects in the intermediate and high-risk PERSEVERE groups who exhibited endotype B, hydrocortisone use was associated with a more than 10-fold reduction in in the risk of death or prolonged MODS. Hydrocortisone use was not associated with clinical outcomes in subjects with endotype A. This suggested that hydrocortisone benefit may be limited to children with a more activated immune state. Our study takes this line of reasoning further and, for the first time, provides evidence of an association between hydrocortisone use and *worse* outcomes in children who have severe functional innate immune impairment. It is therefore possible that prior clinical trials of hydrocortisone use in sepsis may have failed to correctly identify beneficial or harmful effects due to an inability to adjust for subjects’ immunologic state. Since immunoparalysis is typically occult, and biomarkers of immunoparalysis are not currently measured in the clinical laboratory, it will be crucial to include prospective immune phenotyping in the design of future clinical trials of hydrocortisone in septic children.

Additionally, our results showed that receipt of a RBC transfusion was also associated with a higher risk of a longer duration of MODS, an observation that was recently the subject of another secondary analysis of this data set [32]. We also found that the presence of baseline immune compromise was associated with fewer MODS days in patients with immunoparalysis. In this patient population, immune suppression may be a modifiable risk factor through the tapering of immunosuppressive medication. Further, the presence of known baseline immune compromise may prompt earlier and more aggressive sepsis treatment. This is an area of active investigation.

This study was limited by its single center design and small sample size. Despite this, we were able to demonstrate significant associations between hydrocortisone use, immune function, and outcomes. The use of hydrocortisone in this study was not protocolized, and the resulting variability in prescription may have influenced our results. Perhaps most importantly, the majority of subjects who received hydrocortisone in our cohort underwent immune function testing after having received at least one dose of hydrocortisone. It is therefore unclear if hydrocortisone contributed to the development of immunoparalysis or if subjects at high risk for immunoparalysis are also more likely to receive hydrocortisone. The cause-and-effect relationship between hydrocortisone and innate immune suppression in critically ill children is an active area of investigation for our research group. The presence of a differential relationship between hydrocortisone use and outcomes depending on the host immune response, however, remains a key confounder of future clinical trials of hydrocortisone in pediatric sepsis. Lastly, this study focuses primarily on the TNFα response, which represents only one measure of immune function. While other aspects of immune function including phagocytosis, intracellular killing, antigen presentation, adaptive immune responses, and immunologic memory may be of interest in this population, a large body of literature suggests that the TNFα response is a highly clinically relevant readout of immune function in septic children.

## Conclusion

The administration of hydrocortisone to children with immunoparalysis in the setting of severe sepsis/septic shock was associated with a longer duration of multiple-organ dysfunction syndrome whereas this association was not seen in children without immunoparalysis. While cause and effect relationships cannot be determined from this study, these data highlight the need to include prospective immunophenotyping in clinical trials of hydrocortisone in septic children.

## Data Availability

The datasets used and/or analyzed during the current study are available from the corresponding author on reasonable request.
